# Modeling the diagnosis of coronary artery disease by discriminant analysis and logistic regression: a cross-sectional study

**DOI:** 10.1186/s12911-022-01823-8

**Published:** 2022-03-29

**Authors:** Sahar Shariatnia, Majid Ziaratban, Abdolhalim Rajabi, Aref Salehi, Kobra Abdi Zarrini, Mohammadali Vakili

**Affiliations:** 1 Department of Biostatistics and Epidemiology, Faculty of Health, Golestan University of Medica Science, Gorgan, Iran; 2 Department of Electrical Engineering, Faculty of Engineering, Golestan University, Gorgan, Iran; 3 Health Management and Social Development Research Center, Department of Biostatistics and Epidemiology, Faculty of Health, Golestan University of Medical Sciences, Gorgan, Iran; 4 Ischemic Disorders Research Center, Golestan University of Medical Sciences, Gorgan, Iran; 5 Intensive Care Unit of Fatemeh Zahra Hospital, Mazandaran University Medical Sciences, Sari, Iran; 6 Health Management and Social Development Research Center, Department of Biostatistics and Epidemiology, Faculty of Health, Golestan University of Medical Sciences, Gorgan, Iran

**Keywords:** Coronary artery disease, Discriminant analysis, Logistic regression

## Abstract

**Purpose:**

Coronary artery disease (CAD) is one of the most significant cardiovascular diseases that requires accurate angiography to diagnose. Angiography is an invasive approach involving risks like death, heart attack, and stroke. An appropriate alternative for diagnosis of the disease is to use statistical or data mining methods. The purpose of the study was to predict CAD by using discriminant analysis and compared with the logistic regression.

**Materials and methods:**

This cross-sectional study included 758 cases admitted to Fatemeh Zahra Teaching Hospital (Sari, Iran) for examination and coronary angiography for evaluation of CAD in 2019. A logistics discriminant, Quadratic Discriminant Analysis (QDA) and Linear Discriminant Analysis (LDA) model and K-Nearest Neighbor (KNN) were fitted for prognosis of CAD with the help of clinical and laboratory information of patients.

**Results:**

Out of the 758 examined cases, 250 (32.98%) cases were non-CAD and 508 (67.22%) were diagnosed with CAD disease. The results indicated that the indices of accuracy, sensitivity, specificity and area under the ROC curve (AUC) in the linear discriminant analysis (LDA) were 78.6, 81.3, 71.3, and 81.9%, respectively. The results obtained by the quadratic discriminant analysis were respectively 64.6, 88.2, 47.9, and 81%. The values of the metrics in K-nearest neighbor method were 74, 77.5, 63.7, and 82%, respectively. Finally, the logistic regression reached 77, 87.6, 55.6, and 82%, respectively for the evaluation metrics.

**Conclusions:**

The LDA method is superior to the Quadratic Discriminant Analysis (QDA), K-Nearest Neighbor (KNN) and Logistic Regression (LR) methods in differentiating CAD patients. Therefore, in addition to common non-invasive diagnostic methods, LDA technique is recommended as a predictive model with acceptable accuracy, sensitivity, and specificity for the diagnosis of CAD. However, given that the differences between the models are small, it is recommended to use each model to predict CAD disease.

## Introduction

Cardiovascular diseases are of the leading causes of death worldwide. One of the most critical heart diseases is coronary artery disease (CAD). Coronary arteries include Left Anterior Descending (LAD), Left circumflex (LCX), and Right Coronary Artery (RCA), divided into left main artery (LMA) including LCX and LAD and right coronary artery (RCA). Suffering from CAD means that at least one of these arteries narrowness is more than 50% [1]. Examination and the study of various sources indicate that the risk factors for CAD are smoking, hypertension, hyperlipidemia (high total cholesterol, high triglycerides and high-density lipoproteins (HDL), Low-density lipoprotein (LDL)), diabetes, physical inactivity, obesity, abdominal obesity, unhealthy diet, age, gender, family history of heart disease, alcohol consumption, psychological factors, menopause, acute phase protein, high fasting glucose, fibrinogen, lipoprotein and homocysteine[1–5]. In examining the causes of death in Iran in 2009, it was found that out of 321,570 deaths in this year, 82,307 were due to CAD, which is the first cause of death with 25.6% [6].

Exercise testing, echocardiogram, nuclear scans of the heart and angiography are the ways to diagnose the disease, where angiography is the most accurate way to detect it [7, 8]. In a study on 598,792 patients, the mortality rate after angiography was 0.1% and the total number of major complications was about 1.7% [9]. Despite this angiography is an expensive and invasive procedure and is associated with risks like death, heart attack and stroke [2]. It is widely used to identify the causes of diseases and diagnostic strategies with higher accuracy and fewer side effects. Data mining and machine learning techniques are similar to decision trees, neural networks, Bayesian networks, and support vector machines. [10–12]. Predictions and classifications are a common practice in applied research. Some of the most widely used mathematical methods for predictions and classifications are discriminant analysis [13], logistic regression [14], neural networks [15], and classification and regression trees (CART) [16]. The statistical techniques are mostly divided into two categories, classical and non-classical. In classical statistics, this task is mainly done with the help of methods like regression, discriminant analysis, time series, regression tree and logistic regression, and in non-classical statistics, it is the duty of data mining and machine learning techniques [17–19].

The literature review showed that different algorithms such as clustering, classifications, regression and association rules, decision trees, Bayesian network, neural network, multi-layer perceptron with error back propagation algorithm, scaled conjugate gradient (SCG) and support vector machine (SVM) have been used for predicting CAD [20–32]. However, the comparison between the algorithms has not received adequate attention. Therefore, the purpose of this modelling was to provide insight based on information available from a specific subject.

Given the importance of the issue of prediction prognosis of CAD and the fact that, up to now, no comprehensive comparative study has been conducted in Iran to predict CAD, this study tried to predict the prognosis of CAD using discriminant analysis and compared with the logistic regression model.

The rest of this paper was organized as follows. In Sect. 2 we introduce our data sources and statistical methods we used. In Sect. 3 we presented our results, including sample characteristics, variable selection, and compare the prediction performance of the statistical models. In Sect. 4 we discussed the results. Section 5 includes concluding remarks and a description of directions for future research.

## Methodology

### Experimental sample

This diagnostic study was carried out as cross-sectional. The population study was all the patients with cardiovascular disease who were admitted to Fatemeh Zahra Teaching Hospital, (Sari, Iran) and underwent angiography of coronary arteries. The indication for angiography for patients were clinical indication including chest pain/ chronic coronary syndrome or unstable patients with myocardial infarction with or without ST segment elevation. The sample size in this study, according to the study of Kurt et al. [33] and considering the estimated area under the ROC curve of 75% for different methods and with a precision of 0.05 at 95% confidence level and 80% power was estimated. Also, considering the ratio of negative angiography to positive angiography cases equal to 0.33 (N− = 188 and N+  = 568), 756 samples in PASS11 are estimated for this study. Based on formulas is the following:

1 $$\begin{aligned} & Var\left( {\widehat{AUC}} \right) = V\left( {AUC} \right) = \left( {0.0099*e^{{\frac{{ - \alpha^{2} }}{2}}} } \right)*(6\alpha^{2} + 16) \\ & a = \varphi^{ - 1} ({\text{AUC}})*1.414\quad {\text{AUC}}_{{{\text{H0}}}} = 0.75\quad {\text{AUC}}_{{{\text{H1}}}} = 0.80 \\ & {\text{V}}_{{{\text{H}}_{0} \left( {\widehat{{{\text{AUC}}}}} \right)}} = 0/134842\quad {\text{V}}_{{{\text{H}}_{1} \left( {\widehat{{{\text{AUC}}}}} \right)}} = 0/119422 \\ \end{aligned}$$

2 $$n = \frac{{\left[ {z_{{\frac{\alpha }{2}}} \sqrt {V_{{H_{0} \left( {\widehat{AUC}} \right)}} } + Z_{\beta } \sqrt {V_{{H_{1} \left( {\widehat{AUC}} \right)}} } } \right]^{2} }}{{\left[ {AUC_{1} - AUC_{0} } \right]^{2} }} = 409$$

This study contained the records of 758 patients, each of which has 19 variables. All variables can be considered as indicators of CAD for a patient, according to medical literature [34–38]. The variables are arranged in three groups: demographic, clinical, and laboratory variables (Table 1). Some of the variables in the presented tables should be further explained: current smoker is current consumption of cigarettes, illicit drug abuse is current use of illegal drug use (opium, heroin, etc.), and alcohol consumption is the lifetime use of alcohol. Each patient could be in two possible categories CAD or Normal. A patient is categorized as CAD, if his/her diameter narrowing is greater than or equal to 50%, and otherwise as Normal based on the results of angiography and specialist diagnosis.

**Table 1:** Variables of study dataset

Type variables	Variable name
Demographic	Age
	Gender
	Blood group
	Antigen
	Weight
	Height
	BMI (body mass index kg/m^2^)
	Hypertension history
	Family history of heart disease in first-degree relatives
	History of diabetes
	Smoking
	Illicit drug abuse
	Alcohol consumption
Clinical	Systolic blood pressure
	Diastolic blood pressure
Laboratory	fasting blood sugar (FBS)
	Creatinine (Cr)
	Blood urea nitrogen (BUN)
	Low-density lipoprotein (LDL)
	Triglyceride (TG)
	Total cholesterol (TC)
	High-density lipoprotein (HDL)

The inclusion criteria were patients who were admitted to the hospital for angiography due to cardiovascular disease with indication for angiography, and the exclusion criteria were patients who had received angiography before angiography or received any treatment after angiography. Diagnostic cases of CAD or non-CAD were recorded in the patient's file based on the results of angiography and physician's opinion. The study protocol and experimental protocols was approved by the Ethics Committee of Golestan University of Medical Sciences (IR.GOUMS.REC.1398.031). All methods were carried out in accordance with relevant guidelines and regulations, and a consent form was obtained from all the participants.

### Statistical methods

#### Classical methods

##### Discriminant analysis (DA)

DA is one of the multivariate statistical methods for classifying a set of observations and discriminant analysis is a statistical technique that allows the researcher to distinguish between two or more groups according to several independent variables simultaneously. This technique is one of the multivariate statistical methods used to classify a set of observations as well as assign new observations to predefined categories. In other words, with DA technique, one can combine the linear composition of the independent variables as a discriminant function and divide the observations into two or more categories [39].

DA is one of the oldest and most well-known classification techniques proposed by Ronald Fisher in 1936 and generalized by others in later years [40, 41]. Over the past years, various discriminant functions have been examined, but they were similar in terms of purpose. Common types of parametric DA are linear discriminant analysis (LDA), Quadratic Discriminant Analysis (QDA), Regularized Discriminant Analysis (RDA), and the nonparametric DA is K-Nearest Neighbor (KNN) Analysis method [42].

##### Linear discriminant analysis (LDA)

LDA uses linear combination of independent variables to create the maximum intergroup ratio to intragroup changes in discriminant scores. Among the most popular functions used in DA is the Fisher discriminant function. In another method, the ranking rule is obtained by minimizing the average cost function (ECM) [17].

DA examines the relationship between several independent variables and the class response variable. The easiest type of analysis is when the response variable has two groups. In this case, the discriminant linear function, which passes through the average of the two groups (centers), can be used to separate the two groups, and when there are several prediction groups, k-1, where k is the number of classes, is required for classification. Imagine there are two groups:

If $\overline{{X_{1} }}$ and $\overline{{X_{2} }}$ are the mean of the first and the second groups, respectively, and S is the merged variance–covariance matrix, discriminant function of Fisher does separation as follows:

X_i_ is the member of group one if:

3 $$y = \left( {\overline{{x_{1} }} - \overline{{x_{2} }} } \right)^{\prime } S^{ - 1} X_{i} \ge \frac{1}{2}\left( {\overline{{x_{1} }} - \overline{{x_{2} }} } \right)^{\prime } S^{ - 1} \left( {x_{1} + x_{2} } \right)$$

X_i_ is the member of the second group if:

4 $$y = \left( {\overline{{x_{1} }} - \overline{{x_{2} }} } \right)^{\prime } S^{ - 1} X_{i} < \frac{1}{2}\left( {\overline{{x_{1} }} - \overline{{x_{2} }} } \right)^{\prime } S^{ - 1} (x_{1} + x_{2} )$$

Multivariate normalization, homogeneity of variance–covariance matrix, linearity and the absence of multicollinearity between independent variables are of the assumptions of LDA [43], yet Tabachnick and Fidell showed that the linear discriminant function is robust against the deviation from the multivariate normality due to the presence of outlier data as well as violations of the homogeneity of the variance–covariance matrix [44].

##### Quadratic discriminant analysis (QDA)

QDA is another DA technique that like LDA creates classification functions with independent variables. However, the functions are not linear in QDA. In some cases, linear functions may not create the best group separation, and using Quadratic Discriminant Functions (QDFs) may be more appropriate. However, in selecting the type of model, one must pay attention to the assumptions. Multivariate normal of the variables are independent of QDA method assumptions, but it does not assume variance–covariance homogeneity [45].

Thus, QDA is a more appropriate analysis in case of variance–covariance heterogeneity.

QDA assigns the case i to group one if the following equation holds; otherwise, case i belongs to group two. Here, $\overline{{X_{1} }}$ and $\overline{{X_{2} }}$, respectively, are the mean of the first and the second groups, and S_1_ and S_2_ are variance–covariance matrix of the first and the second groups, respectively.

5 $$\frac{1}{2}x_{i} \left( {S_{1}^{ - 1} - S_{2}^{ - 1} } \right)x_{i}^{\prime } + \left( {\overline{{x_{1} }} S_{1}^{ - 1} - \overline{{x_{2} }} S_{2}^{ - 1} } \right)x_{i}^{\prime } - k \ge \ln \left( {\frac{{c_{12} p_{2} }}{{c_{21} p_{1} }}} \right)$$

where

6 $$k = \frac{1}{2}\ln \left( {\frac{{\left| {S_{1} } \right|}}{{\left| {S_{2} } \right|}}} \right) + \frac{1}{2}\left( {\overline{{x_{1} }} S_{1}^{ - 1} \overline{{x_{1}^{\prime } }} - \overline{{x_{2} }} S_{2}^{ - 1} \overline{{x_{2}^{\prime } }} } \right)$$

Consider the Mahalanobis distance (MD). This function is as follows:

7 $${\text{f}}\left( {\text{x}} \right) = {\text{D}}_{1}^{2} \left( {\text{x}} \right) - {\text{D}}_{2}^{2} \left( {\text{x}} \right) + {\text{ln}}\left( {\frac{{\left| {S_{1} } \right|}}{{S_{2} }}} \right) - 2{\text{ln}}\left[ {\left( {\frac{{{\text{p}}_{2} }}{{{\text{p}}_{1} }}} \right)} \right]$$

The $\mathrm{f}\left(\mathrm{x}\right)={\mathrm{D}}_{1}^{2}\left(\mathrm{x}\right)-{\mathrm{D}}_{1}^{2}\left(\mathrm{x}\right)$ value of MD is to the second power when ${S}_{1}\ne {S}_{2}$ decreases to the linear function [39, 46]. As the assumption of multivariate normality and homogeneity of covariance variance matrix is not established in this study, various types of DA were used.

##### Logistic regression (LR)

LR is the commonest method used to examine the relationship between independent variables and qualitative response variables, especially the dichotomous response variable. LR is a multivariate statistical method where the dependent variable (response) is a nominal or ranked variable, and the independent variable or variables can be continuous, discrete, nominal, or ranking [47].

In LR, independent variables that can be continuous or discrete are used. This model is basically used to identify the relationship between two or more independent and dependent variables. LR model is for *P*_i_ (probability of response) to n independent variables X_1_, X_2_,… and X_n_. The name “Logistics” is derived from the word “Logit,” which is actually a variable change as follows.

8 $$logit\left({P}_{i}\right)=\mathrm{log}\left(\frac{{P}_{i}}{1-{P}_{i}}\right)={\beta }_{0}+{\beta }_{1}{X}_{1}+\dots +{\beta }_{n}{X}_{n}$$

In other words, Logit is a probability equal to the log of the ratio of chance or fraction whose numerator is the probability of an accident and its denominator is the probability of an accident. Converting P to $ln\frac{{P}_{i}}{1-{P}_{i}}$ causes the range of changes in p, which is from zero to one, to be from − ∞ to + ∞. *β*_i,s_ are the parameters estimated and *P*_i_ are the probability of the response. In using logistic regression to predict CAD, β_o_ is the y-intercept and *β*_i_ coefficients of the independent variables [48].

In the data analysis, while examining the assumptions, to fit the discriminant analysis model, its assumptions were examined, and the results showed that according to the Mardia's Skewness test, the multivariate normality assumption is not established due to high Skewness and according to Breusch-Pagan test, variance–covariance matrix homogeneity assumption was not established in the two groups either. Tolerance and Variance Inflation were used to examine the multicollinearity between the independent variables. As the tolerance values were less than 0.1 and Variance Inflation less than 10, multicollinearity assumption was not violated. In this case, given the capability of DA, LDA techniques, QDA, discriminant analysis with KNN were used and compared, the value of k = 15 was obtained using cross-validation method in KNN.

In order to fit the logistic regression model, the Hosmer–Lemeshow test was used to confirm the fit of the model, indicating that the logistic regression model can be used for this data set. In addition, the stepwise LR test is used to determine the predictor variables and perform multivariate analysis to adjust the effect of the variables. Odds Ratio (OR) and 95% confidence interval (95% CI) were calculated for the variable and the p. values less than 0.05 were considered as statistically significant. For model goodness of fit and to determine the accuracy, we used the diagnostic test indicators of receiver operating characteristics (ROC), accuracy, sensitivity and specificity. The statistical software package SAS (Statistical Analysis System) was used to perform a variety of DA and logistic regression.

#### Non-classical method

##### K-nearest neighbour (KNN) analysis

KNN is the first nonparametric DA presented by Fix and Hodges in 1951 [49, 50]. This analysis does not consider the normality of several variables, but assumes homogeneity of variance. KNN differs from the above methods. It does not find functions for group differentiation, but classifies observations based on group membership of K value [51].

The distance between the two observations is calculated from the following equation:

9 $${d}^{2}\left({x}_{1},{x}_{2}\right)={\left({x}_{1}-{x}_{2}\right)}^{^{\prime}}{V}^{-1}({x}_{1}-{x}_{2})$$

## Results

### Sample characteristics

Of the 758 subjects with suspected CAD who underwent coronary angiography, 409 (54%) were males and 349 (46%) females. The mean age and standard deviation were 59.11 ± 11.11 years with the lowest and highest age 27 and 90 years, respectively. Moreover, 250 (32.98%) subjects were non-CAD and 508 (67.02%) subjects were diagnosed with CAD.

Results of univariate logistic regression model suggested that the gender of men is associated with a higher risk of CAD (OR_men vs women_ = 3.50, 95% CI: 2.55–4.82). Smoking (OR_yes vs no_ = 2.63, 95% CI:1.61–4.29) and illicit drug abuse (OR_yes vs no_ = 3.50, 95% CI:1.96–6.34) were similarly associated with the risk of CAD. Age (OR = 1.06, 95% CI: 1.04–1.08), FBS (OR = 1.005, 95% CI: 1.002–1.009), BUN (OR = 1.07, 95% CI: 1.04–1.11), Cr (OR = 6.56, 955 CI: 3.02–14.26), systolic blood pressures (OR = 1.02, 95% CI: 1.01–1.03) and diastolic blood pressures (OR = 1.02, 95% CI: 1.008–1.03) were directly associated with a greater risk of CAD. Conversely, according to the results, body mass index (OR = 0.91, 95% CI: 0.88–0.94) and HDL (OR = 0.96, 95% CI: 0.94–0.98) associated with a lower risk of CAD (Table 2).

**Table 2:** Univariate logistic regression the association of independent variables with coronary artery disease

Parameters	Coefficient (β)	S.E(β)	OR CI (0.95%)	P-value
Gender				
Female	Ref		Ref	
Male	1.25	0.16	3.50 (2.55–4.82)	< 0.001
Smoking				
No	Ref		Ref	
Yes	0.96	0.24	2.63 (1.61–4.29)	< 0.001
Illicit drug abuse				
No	Ref		Ref	
Yes	1.26	0.29	3.50 (1.96–6.34)	< 0.001
Blood group				
A	− 0.15	0.18	0.85 (0.59–1.23)	0.40
B	− 0.07	0.19	0.93 (0.62–1.37)	0.71
AB	0.03	0.32	1.03 (0.54–1.96)	0.90
O	Ref		Ref	
Antigen				
Negative	Ref		Ref	
Positive	0.31	0.29	1.36 (0.76–2.41)	0.28
History of Blood pressure				
No	Ref		Ref	
Yes	0.13	0.15	1.14 (0.84–1.56)	0.37
Family history of heart disease				
No	Ref		Ref	
Yes	0.37	0.20	1.46 (0.97–2.18)	0.06
History of Blood pressure				
No	Ref		Ref	
Yes	0.18	0.18	1.20 (0.84–1.72)	0.30
Alcohol use				
No	Ref		Ref	
Yes	0.81	0.55	2.25 (0.75–6.74)	0.14
Age	0.06	0.008	1.06 (1.04–1.08)	< 0.001
BMI	− 0.09	0.01	0.91 (0.88–0.94)	< 0.001
FBS	0.005	0.001	1.005 (1.002–1.009)	0.001
TC	0.001	0.001	1.001 (0.99–1.00)	0.33
TG	− 0.000	0.000	0.99 (0.99–1.00)	0.14
LDL	0.004	0.002	1.00 (0.99–1.00)	0.05
HDL	− 0.03	0.009	0.96 (0.94–0.98)	< 0.001
BUN	0.07	0.01	1.07 (1.04–1.11)	< 0.001
Cr	1.88	0.39	6.56 (3.02–14.26)	< 0.001
Systolic blood pressure	0.02	0.005	1.02 (1.01–1.03)	< 0.001
Diastolic blood pressure	0.02	0.007	1.02 (1.008–1.03)	0.002

The results of multivariate logistic regression analysis with step-by-step variable entry technique led to the elimination of the significance of the diastolic blood pressure, BUN, Cr, and smoking. According to the results, the risk of CAD in males was 4 times higher than in females (OR: 4.01, 95% CI: 2.67–6.01), in people with illicit drug abuse were 2.17 times higher than others (OR: 2.17, 95% CI: 1.14–4.13), and in families with a history of cardiovascular disease were 1.93 times higher than in families without a history (OR: 1.93, 95% CI: 1.20–3.09) (Table 3).

**Table 3:** Multivariate logistic regression the association of independent variables with coronary artery disease

Parameters	Coefficient (β)	S.E(β)	OR CI (0.95%)	*P* value
Intercept	− 6.21	1.23	0.002	< 0.001
Gender				
Female	Ref	–		
Male	1.39	0.21	4.01 (2.67–6.01)	< 0.001
Family history of heart disease				
No	Ref	–		
Yes	0.66	0.24	1.93 (1.20–3.09)	0.006
Illicit drug abuse				
No	Ref	–		
Yes	0.78	0.33	2.17 (1.14–4.13)	0.019
Age	0.08	0.01	1.08 (1.06–1.10)	< 0.001
BMI	− 0.05	0.02	0.95 (0.91–0.99)	0.016
FBS	0.007	0.002	1.007 (1.003–1.011)	< 0.001
HDL	− 0.035	0.012	0.96 (0.94–0.99)	0.004
LDL	0.013	0.003	1.013 (1.007–1.019)	< 0.001
Systolic blood pressure	0.018	0.006	1.018 (1.007–1.03)	0.001

### Models evaluation

The comparison results of the evaluation indicators showed that the accuracy of LDA was 78.6% and 1.8% higher than that of the logistic regression method. The QDA model was the highest sensitivity, 88.2%, and the KNN has the lowest sensitivity, 77.5%. In terms of specificity, QDA with 48.2% and LDA with 71.3% were estimated as the most and least in the models, respectively (Table 4).

**Table 4:** The accuracy, sensitivity, specificity and AUC models

Methods	Model	Accuracy %	Sensitivity %	Specificity %	AUC %
Classical	LDA	78.6	81.3	71.3	81.9
	QDA	64.6	88.2	48.2	81
	LR	77	87.6	55.6	82
Non-classical	KNN	74	77.5	63.7	82

*LDA* linear discriminant analysis, *QDA* quadratic discriminant analysis, *KNN* K-nearest neighbor, *LR* logistic regression

**Fig. 1:**
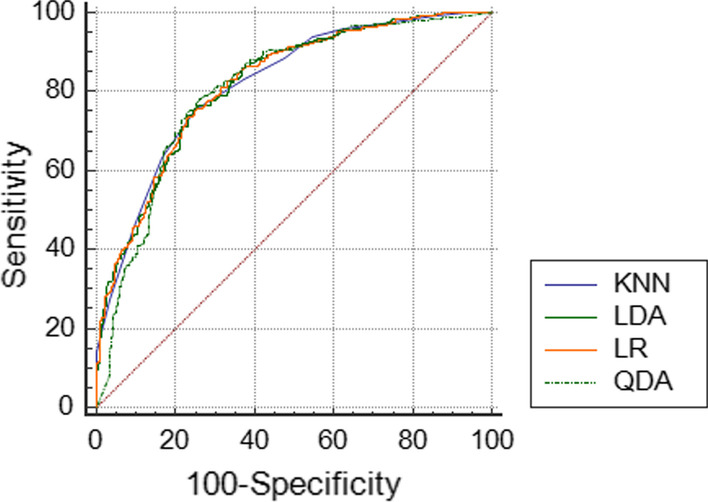
ROC curve for logistic regression models, linear discriminant analysis, quadratic discriminant analysis, and K nearest neighbor

The ROC curves of the four models clearly indicated that the models are similar together, with no difference in the area under the curve (AUC). The level below the ROC curve in four modelling techniques was very close and estimated to be between 81 and 82%. According to the Delong test [52], there was no significant difference in the level below the ROC curve of the model when the AUC values of the techniques are compared pairwise (Fig. 1 and Table 5).

**Table 5:** Differences in surface area under the ROC curve in linear discriminant analysis, quadratic discriminant analysis, K nearest neighbor and logistic regression

Two by two comparison	Difference between areas	Standard error	95% CI	z statistic	*P*-value
KNN					
LDA	0.0009	0.011	(− 0.021 to 0.02)	0.782	0.93
LR	0.00003	0.011	(− 0.023 to 0.023)	0.002	0.99
QDA	0.01	0.014	(− 0.017 to 0.039)	0.73	0.46
LDA					
LR	0.0008	0.001	(− 0.001 to 0.0036)	0.61	0.54
QDA	0.009	0.014	(− 0.01 to 0.03)	0.69	0.49
LR					
QDA	0.01	0.01	(− 0.016 to 0.037)	0.75	0.44

*LDA* linear discriminant analysis, *QDA* quadratic discriminant analysis, *KNN* K-nearest neighbor, *LR* logistic regression

In general, all models converged in similar results. All methods estimated the same statistically significant coefficients. The overall classification rate for all was good, and either can be helpful in classifying the class membership of CAD. LDA slightly exceeds discriminant function in the correct classification rate but when taking into account sensitivity, specificity and AUC the differences in the AUC were negligibly, thus indicating no discriminating difference between the models.

Accuracy, sensitivity, specificity and AUC indices of LR Models, LDA, QDA and KNN presented in Table 5 and Fig. 1.

## Discussion

Cardiovascular disease is the most important cause of death in Iran and the rest of the world. The most important recent epidemiological study of the Iranian population indicates that CAD is common among young people in this population [53]. Cardiovascular disease is usually caused by a combination of multiple risk factors. It has been clearly shown that changes in risk factors can reduce mortality, especially in people with unknown cardiovascular diseases[54].

We found that age, gender, illicit drug abuse, family history of heart disease, systolic blood pressure, as well as FBS, HDL, LDL, and BMI all play an important role in CAD risk. So that, in the study of Bidel et al. [55], gender, smoking, family history of first-degree relatives, systolic blood pressure, diastolic blood pressure, and high-density lipoprotein were significantly associated with CAD. Also, in the study of Sut et al. [56], age, gender, cholesterol, triglyceride, low-density lipoprotein, high-density lipoprotein, a history of diabetes, and a history of smoking were significantly associated with CAD. The results of those studies are almost consistent with our findings. The most significant feature discovered by logistic regression is illegal drug use. Illicit drug abuse has been identified as a "risk factor" for an association between Illicit drug abuse and a high risk of coronary artery disease. However, limited research has indicated that this relationship may differ depending on the type of region and the type of drug used in that region [57–60]. Another finding of logistic regression is that BMI is related to coronary artery disease. However, this relationship was inverse, so that with increasing body mass index, the risk of coronary artery disease decreases. This finding may seem to contradict the findings of other studies [34, 38]. However, it should be noted that the subjects were not all healthy individuals but those who referred for angiography with a complaint of heart disease and their coronary artery disease was confirmed by angiography and the other group was not confirmed. However, people who are not approved are still at risk for heart disease and may have a higher body mass index.

Subsequently, we report on a study in which we developed several predictive models to predict CAD. In particular, we used LR, KNN, LDA, and QDA. In addition, we evaluated the performance of the model based on SEN, SPE, Accuracy, and AUC. We tested the statistical significance of the difference in the area under the ROC curve in pairwise. Comparison of the results of evaluation indices showed that the accuracy of LDA was higher than logistic regression. In terms of sensitivity indicators, QDA is the highest and KNN is the lowest. In addition, their AUCs are very close in almost all models.

According to the literature review, numerous studies have been conducted to predict CAD by using diagnostic models. In the study of Dwivedi et al. [61], data mining methods were used to predict CAD disease. Among these methods, the accuracy and sensitivity of KNN were more than LR, which is in line with the present study. However, the specificity of the logistic regression model is higher than that of KNN, which is inconsistent with this study. In addition, the study by Sut et al. [56] Shows that when LR and QDA models are used to diagnose CAD, QDA is more accurate. This is consistent with the results of this study. In addition, the study conducted by Antonogeorgos et al. [62] evaluated variables related to asthma. Among them, the accuracy and specificity of LR and the sensitivity of DA are higher, which is inconsistent with this study. But in terms of the area below the ROC curve, the LR method performed better, which is in line with the present study. Also, Sadehi et al. [17] showed in their metabolic syndrome prediction study that the area under the ROC curve of the LR method was higher than that of the LDA, and the accuracy of the LDA was higher than LR, which is consistent with this study. In addition, the study of Alizadeh et al. with purpose evaluated the coronary artery disease detection using Support Vector Machine (SVM) showed that accuracy rates of 86.14%, 83.17%, and 83.50% were achieved for the diagnosis of the stenosis of the left anterior descending (LAD) artery, left circumflex (LCX) artery and right coronary artery (RCA), respectively, indicating the best performance [63]. In another study, Kurt et al. [33] employed a logistic regression on a dataset. The results showed that accuracy of classification technique was assessed using ROC curve, the logistic regression (LR) was 0.753, indicating the low accuracy compared to our study. Also, Colombet et al. [64] showed in their cardiovascular risk prediction study that the area under the ROC curve of the LR method was 0.78 (0.75–0.81), which indicate the low accuracy compared to our study.

We suggest that gender, family history of heart disease, illicit drug abuse, age, BMI, FBS, HDL, LDL and Systolic blood pressure variables may be used as reliable indicators to predict presence of CAD. In our study, we compared methods by using a real data set in order to provide information on general tendency of data structures in data sets and help researchers to select best method for solving problems of classification. On the basis of these considerations, the linear discriminant analysis method is superior to the QDA, KNN and LR methods in differentiating CAD patients. Therefore, in addition to common non-invasive diagnostic methods, LDA technique is recommended as a predictive model with acceptable accuracy, sensitivity, and specificity for the diagnosis of CAD.

There are a few limitations with this study. First, this was a cross sectional study design with the documented data that abnormalities in biologic characteristics such as laboratory values we do not know whether they preceded the disease or were a result of the disease. Second limitation, we only collected data from one hospital which may limit the generalizability of the developed models, so data collection from more hospitals or population based should be considered. We also need more data from patients e.g., clinical symptoms and electrocardiogram (ECG). However, consistent with the purpose of the current research, considering only the routine clinical features of the patients while being admitted would suffice. Another limitation of our study was the low number of samples to compare different models.

## Conclusion and future work

In this study, several models were applied on the dataset and the results were discussed. The variables included in this dataset are possible indicators of CAD, according to our medical knowledge. The accuracy value achieved in this study is, to the best of our knowledge, higher than currently reported values in the literature.

In future, we aim to consider predicting state of each artery independently. Moreover, it is obvious that true diagnosis of diseased people is more important than true identification of healthy ones. Finally, larger datasets, more variables and also broader data mining approaches, could be used to achieve better and more interesting results, and these models need to be compared with the artificial neural network and machine learning models as well.

## Data Availability

The data that support the findings of this study are available from Golestan University of Medical Sciences but restrictions apply to the availability of these data, which were used under license for the current study, and so are not publicly available. Data are however available from the authors upon reasonable request and with permission of Golestan University of Medical Sciences.
